# Salinity as an Inducer of Antioxidant Activity Exerted by Mangrove Species from Campeche, Mexico

**DOI:** 10.3390/plants14050800

**Published:** 2025-03-04

**Authors:** Carlos A. Chan-Keb, José L. Aragón-Gastélum, Claudia M. Agraz-Hernández, Román A. Pérez-Balan, Eduardo J. Gutiérrez Alcántara, Marco A. Popoca-Cuaya, Mónica A. Guillen-Poot, Emanuel Hernández-Núñez, Francisco J. Aguirre-Crespo

**Affiliations:** 1 Facultad de Ciencias Químico-Biológicas, Universidad Autónoma de Campeche, San Francisco de Campeche, Av. Agustín Melgar S/N entre Calle 20 y Juan de la Barrera, Col. Buenavista, Campeche CP 24039, México; carachan@uacam.mx (C.A.C.-K.); jlaragon@uacam.mx (J.L.A.-G.); roaperez@uacam.mx (R.A.P.-B.); ejgutier@uacam.mx (E.J.G.A.); mapopoca@uacam.mx (M.A.P.-C.); 2 Instituto EPOMEX, Universidad Autónoma de Campeche, San Francisco de Campeche, Av. Agustín Melgar S/N entre Calle 20 y Juan de la Barrera, Col. Buenavista, Campeche CP 24039, México; clmagraz@uacam.mx; 3 Independent Researcher, Mérida CP 97215, México; moniguillen7@gmail.com; 4 Departamento de Estudios de Posgrado e Investigación del Instituto Tecnológico Superior del Calkiní en, el Estado de Campeche (ITESCAM), Av. AH Canun S/N San Felipe, Calkiní, Campeche CP 24900, México; ehernandez@itescam.edu.mx

**Keywords:** *Rhizophora mangle*, *Avicennia germinans*, *Laguncularia racemosa*, antioxidant, DPPH

## Abstract

The mangrove ecosystem is reported to have a large diversity of species that develop in environments with high salinity levels. Plant species from mangroves are used in traditional medicine and are potential sources of chemicals entities with therapeutic applications. The present work aims to assess and document the influence of salinity on the antioxidant activity exerted by extracts of mangrove species through spectroscopic and chemical analysis. The highest salinity is recorded in Río Verde (RV) in Petén Neyac (PN), an LPBR site. The leaves of *Laguncularia racemosa* (from RV and PN) recorded the highest extraction yield (35.29 ± 0.45%). Phytochemical analysis indicated the presence of several families of secondary metabolites in the leaves of *Rhizophora mangle*, *Avicennia germinans*, and *L. racemosa* collected in PN and RV, and the chromatographic profile confirms the complexity of the extracts, especially in *L. racemosa*-RV. The highest content of chlorophylls, carotenoids, and simple phenols was recorded in *R. mangle* (in RV and PN); flavonoids were high in *A. germinans* (RV), and the highest antioxidant activity was recorded in *L. racemosa* (RV) using the DPPH model (EC_50_: 39.74 ± 0.91 μg/mL; E_max_: 67.82 ± 1.00%). According to HPLC, gallic acid (GA), and quercetin (Q) are important metabolites in *L. racemosa.* FTIR spectra can identify several chemical groups and fingerprint regions in complex mixtures, such as methanolic extracts of the species under study. In this context, this is the first report on chemical changes resulting from species collected at sites with different degrees of salinity. GA is the main metabolite affected by salinity and participates in the antioxidant activity exerted by the original extract, which could explain the physiological adaptations of *L. racemosa* and its traditional uses. *L. racemosa* (RV) is ideal for a bioguided phytochemical study that would yield valuable knowledge about its medicinal properties, support ecological conservation, and drive innovation across multiple industries. Further analytical studies are needed to corroborate the impact of salinity on the biosynthesis of secondary metabolites.

## 1. Introduction

Mangroves are communities of global distribution [1], are reported on the coastline of several countries, and their forest plays a crucial role in protecting against erosion and saline intrusion; they are a natural barrier against hurricanes and are a feeding area and refuge for endemic and migratory birds and fish [2]. Mangroves are a salt-tolerant group, mainly arboreal, and salinity plays a role in their growth and survival. Diverse studies document that salinity delays propagule growth, and development [3,4].

Taxonomic, morphological, ethnobotanical, phytochemical, and pharmacological aspects of 84 mangrove species have been documented, and 27 species have traditional medicinal uses that require pharmacological validation [5]. Some mangrove species are being studied in the search for molecules with health applications [6]. In Mexico, few studies have investigated the effects of salinity on the metabolic profile or the pharmacological activity induced by crude mangrove extracts. This research becomes particularly relevant due to the significant variability of salinity in Mexican mangroves, which is influenced by the heterogeneity of hydro-sedimentary conditions, topography, and geomorphology of coastal areas, as well as by the diversity of climates [7].

**Figure 1:**
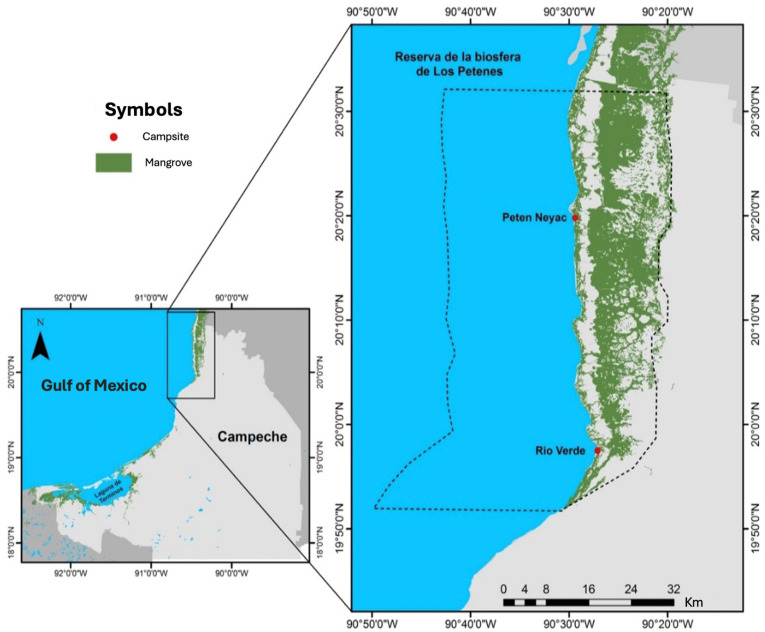
RBLP, Campeche, Mexico. Río Verde (RV; 19°57′27.932″ N; 90°27′4.265″ W) and Petén Neyac (PN; 20°19′48.745″ N; 90°29′23.164″ W) site colect are marked in •.

In Mexico, 25% (194,190 ha) of the mangrove coverage is in Campeche state. This coverage is distributed across the “Área Natural de Protección de Flora y Fauna Laguna de Términos”, and “Río Champotón e Icahao”, and the “Reserva de la Biosfera Los Petenes (RBLP)”. The RBLP is in the northwest coastal strip of the Yucatan Peninsula (Figure 1), and includes “Reserva de la Biosfera Ría Celestún” and the “Área Natural Protegida Estatal El Palmar”. The RBLP is highly significant due to the presence of the Petenes ecosystem, characterized by semi-deciduous forests and mangroves, or solely mangroves, along with its high biodiversity. As a result, it was designated a RAMSAR site in 1999 [8].

The red (*Rhizophora mangle* L.; Rhizophoraceae), black (*Avicennia germinans* L. (L.); Acanthaceae) and white mangrove (*Laguncularia racemosa* (L.) Gaertn f.; Combretaceae), as well as the buttonwood (*Conocarpus erectus* (L.); Combretaceae), are considered the dominant species of the mangrove ecosystem [9]. These species are affected by the modification of water flow, deforestation, organic pollution, solid waste, and chemical pollution, among other factors [10].

*R. mangle*, *A. germinans*, and *L. racemosa* are recognized as resources for the Mayan community settled in the RBLP [11]. However, few studies estimate the physiological, ecological, economic, or medicinal value of secondary metabolites derived from mangrove species [12]. From an ethnomedical perspective, the roots, bark, and leaves of *R. mangle* are used to treat diarrhea, dysentery, leprosy, tuberculosis, and fungal infections as an antiseptic, toothache, bone fractures, and diabetes; pharmacological studies indicate antiulcer and antioxidant activity. Tannins, triterpenes, flavonoid glycosides, quercetin, myricetin, and kaempferol diglycosides have been reported in the *R. mangle* leaves [5,12]. The bark, leaves, and fruits of *A. germinans* are used as an astringent and in the treatment of rheumatism, swelling, throat ailments, diarrhea, bleeding, tumors, swellings, incontinence, sore throats, chest pain, and mouth ulcers; pharmacological studies indicate antimicrobial effects against *Escherichia coli*, *Klebsiella* sp., *Proteus* sp., and *Staphylococcus aureus*. Finally, only the presence of glycosides has been reported [5]. *L. racemosa* has been used as a healing agent, soap, herbicide, and antihypertensive agent [12,13]. Amino acids, polysaccharides, sugars, triterpenes [14], flavonoids, hydrolyzable tannins, condensed tannins, hydrocarbons and fatty acids, sterols, triterpenes, and anthraquinones, as well as simple and complex polyphenols, have been reported in these species [15,16,17,18,19,20,21,22].

In this context, the present work aims to generate chemical, phytochemical, and pharmacological basis to support prospective studies of the leaves of *R. mangle*, *A. germinans*, and *L. racemosa* grown in the Río Verde and Petén Neyac areas of the RBLP. These sites are subject to different environmental conditions that could influence the biosynthesis of the secondary metabolites that are of interest in relation to health.

## 2. Materials and Methods

In Campeche, Mexico, the mangrove ecosystem of the RBLP exhibits the highest degree of conservation. Río Verde (RV; 19°57′27.932″ N; 90°27′4.265″ W) features a coastal forest dominated by *R. mangle*, with *L. racemosa*; beyond this lies a monospecific forest of *A. germinans* (inland forest). Petén Neyac (PN; 20°19′48.745″ N; 90°29′23.164″ W) is also dominated by *R. mangle* along the coastal fringe, with a mixed forest further inland dominated by *R. mangle*, *L. racemosa*, and *A. germinans*. Both sites were selected for their differing hydrological behaviors and health conditions (Figure 1).

### 2.1. Soil Interstitial Water Salinity

The interstitial water salinity of the mangrove forest was measured in situ and obtained from piezometers (PVC; h1 = 1.5 m; h2: 0.5 m depth; h3: 30 cm (maximum root biomass); 0.1 m i.d.; h2: 30 cm; slot 1 cm i.d.c/0.04 × 0.03 m) with stabilized drainage. For this, a refractometer (ATAGO, Inc., Bellevue, WA, USA; 1 mL; range: 0 to 100 Practical Salinity Units (PSU: 1 g/Kg) was used [13].

### 2.2. Collection of Plant Material

Specimens weighing 500 g each of *R. mangle*, *A. germinans*, and *L. racemosa* leaves from mature trees were collected from the RV and PN (September 2018) sites. The specimens were identified as described by Agraz-Hernández et al. [23]. The aerial part of each species was dried at room temperature; subsequently, the dried material was ground and stored [24].

### 2.3. Extraction

Two grams of ground leaves of *R. mangle*, *A. germinans*, and *L. racemosa* (from RV and PN, respectively) were extracted three times (for 24 h each) by cold maceration with 40 mL of methanol at 25 °C [24]. Subsequently, the extracts were filtered, dried under reduced pressure, and stored at 5 °C. Variations in yield were determined by gravimetry.

### 2.4. Qualitative Phytochemistry

The presence of the families of secondary metabolites was assessed as follows: polyphenols (FeCl_3_ test), flavonoids (Shinoda test), tannins (gelatin precipitation), sterols, pentacyclic triterpenes (Liebermann–Burchard test), saponins (foam test), coumarins (fluorescence), alkaloids (Meyer and Dragendorff test), cyanogenic glycosides (Guignard test), cardiotonic glycosides (Keller–Kilani test), and anthraquinones (Bontrager test) [25,26]. For these tests, a stock solution (1 mg/mL) was employed,

### 2.5. Spectroscopic Analysis

Methanolic extracts (1 mg/mL) were examined by UV-Vis spectroscopy (λ = 300–700 nm; Δ = 10 nm) [27]. In addition, 5 mg of extract in 195 mg of KI was employed to develop the FTIR (λ = 4000–500 cm^−1^) spectrum [28]. Functional group and fingerprint regions analyses were used to identify changes in the extracts’ metabolic content and to identify secondary metabolites.

### 2.6. Chromatographic Profile

Under certain modifications, phenols and flavonoids were identified via HPLC according to the method described by Cu-Quiñones [29]. For this, Dionex UltiMate 3000 (obtained from RS HPLC Systems) was used with a mobile phase solution-A (H_2_O-acetic acid 1%), and solution-B (ACN 100%); the chromatography methodology was as follows: 10–40% (0–28 min): 40–60% (28–39 min); 60–90% (39–50 min), and UV detector (λ = 272 nm [30]). Automatic sample injection (20 μL loop), and Chromeleon as a system manager were employed. Separation was achieved by phase reverse column (Alltima HP, C-18 HL, 5 μm *p*.s., i.d. 4.6 × 250 mm). The gallic acid (GA), quercetin (Q), and sample stock solutions (1 mg/mL) were prepared with HPLC-grade H_2_O, followed by sonication for 10 min, and the resulting volume was made up to 1 mL with Sol A. All samples were filtered through a 0.45 μm PVDF-syringe filter. The mobile phase was degassed before injection.

### 2.7. In Vitro Test

From a stock solution (1 mg/mL), chlorophylls and carotenoid content were estimated by spectrophotometry [31]. Simple phenols equivalent to GA were estimated using Folin–Ciocalteu reagent [32]. Flavonoids equivalent to Q were measured using AlCl_3_ 10% solution [33]. The antioxidant activities induced by *R. mangle*, *A. germinans*, and *L. racemosa* extracts (10–560 μg/mL), as well as Q, GA, and caffeic acid (CA) (0.032–32 μg/mL), were determined using the DPPH model [34]. The methanolic extract of *Camellia sinensis* (1–56 μg/mL) was used as a positive control.

### 2.8. Statistical Analysis

In all cases, three independent experiments were carried out with three replicates. The normality of the variables was evaluated with the Shapiro–Wilk method and Levene’s test of homogeneity of variance (α = 0.05). In cases of non-compliance with the assumption of normal distribution, the Box–Cox transformation method was used [35]. The variables were evaluated using a 2-way ANOVA. Finally, a simple linear regression analysis was performed between salinity (independent variable) and phenol concentration (dependent variable). Analyses were performed with STATISTICA V.12 (©Copyright StatSoft, Inc., Palo Alto, CA, USA, 1984–2014) and SPSS 15.0 for Windows (Copyright © 2006 of SPSS Inc., Chicago, IL, USA).

## 3. Results

Higher salinity was recorded in RV than in PN. Salinity increases from the coastal fringe forest where *R. mangle* develops (42.12 and 42.25 PSU) towards the inland forest that houses *A. germinans* (74.87 and 58.93 PSU). Salinity decreases at both sites in forests where *L. racemosa* trees are present (43.25 and 40.59 PSU). Regarding the leaf extraction with methanol, the qualitative phytochemistry of *A. germinans*, *R. mangle*, and *L. racemosa* extracts indicated the presence of alkaloids, saponins, cardiac and cyanogenic glycosides, simple polyphenols, anthraquinones, and flavonoids; likewise, it was recorded that the presence of these metabolic families does not vary according to the collection site. The extracts from leaves of *L. racemosa* collected in RV and PN recorded the highest extraction yields (35.2 ± 0.8 and 35.4 ± 0.1%), compared to *R. mangle* and *A. germinans*, showing significant differences between species (F_2,13_ = 29.08; *p* < 0.0001).

**Figure 2:**
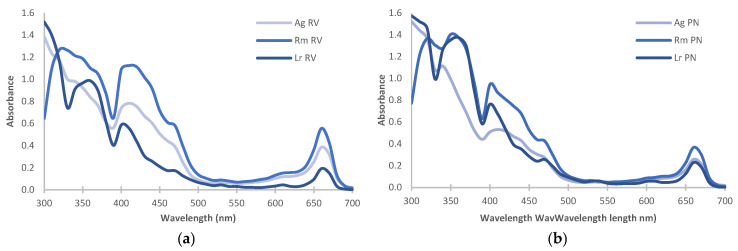
UV-Vis spectra of the methanolic extracts from leaves of *A. germinans*, *R. mangle*, and *L. racemosa* collected in Río Verde (**a**) and Petén Neyac (**b**).

The estimation of chlorophylls (chlorophyll a, chlorophyll b, and total chlorophyll) and carotenoids by UV-Vis spectroscopy is presented in Table 1; the highest pigment content was recorded in *R. mangle.* Similarly, UV-Vis spectroscopy allowed us to establish that RV extracts register hyperchromic effects in the λ: 640–690 nm region, and hypochromic effects (λ: 400–450 nm) were documented in PN extracts (Figure 2).

**Figure 3:**
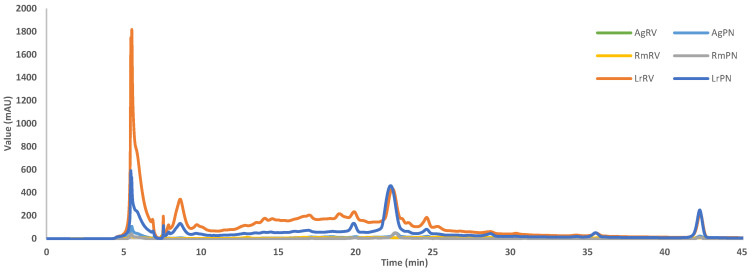
Chromatogram profile obtained by HPLC of the methanolic extracts from *L. racemosa* (Lr), *R. mangle* (Rm), and *L. racemosa* (Lr) leaves collected in Los Petenes Biosphere Reserve. (RBLP; Río Verde (RV) and Petén Neyac (PN)) at λ = 272 nm. Mobile phase: Sol A (H_2_O-acetic acid 1%), Sol B (ACN 100%); 10–40% (0–28 min): 40–60% (28–39 min); 60–90% (39–50 min). Flux: 0.5 mL/min.

To expand the information about the metabolic content of the species, the chromatographic profile indicated that the extracts of *L. racemosa* showed the greatest metabolic diversity, recording the highest number of peaks (*n* = 17; AUC = 2253.26; 50.61%); the most intense peaks were at 5.48, 8.64, 17, and 22.32 min (33.52% AUC; Figure 3). The chromatograms of *A. germinans* and *R. mangle* demonstrated a lower abundance of peaks and low-intensity peaks. The HPLC analysis (λ = 272 nm) indicated that GA and Q peaked at 5.48 and 24.6 min, respectively (Figure S1); however, the presence of other flavonoids was not ruled out [30]. The AUC showed that GA was 8.45 times more abundant than Q (AUC = 665.14 vs. 78.7) in *L. racemosa*-RV extract; this sample had 3.17 and 2.27 times more GA and Q than *L. racemosa*-PN extract. Finally, caffeic acid was related to the peaks located between 15 and 17 min [30].

**Figure 4:**
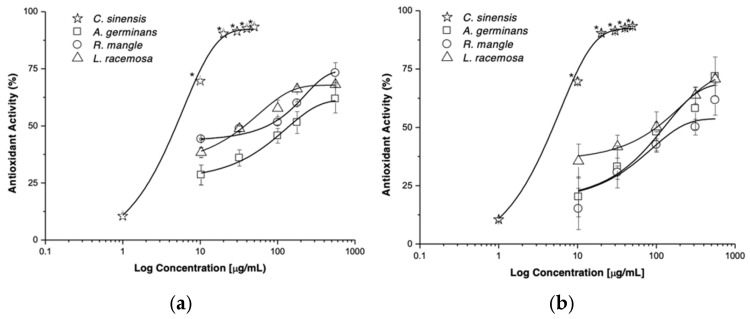
Antioxidant activity by extracts of *L. racemosa*, *R. mangle*, and *A. germinans* collected in Río Verde (**a**) and Petén Neyac (**b**) of the RBLP. The results are the average ± SD of three experiments with three replicates. (* *p* < 0.05).

Additionally, the presence of phenols and flavonoids did not register significant variations between species or between collection sites (Table 2). On the other hand, a positive correlation was recorded between phenols content and salinity at both the PN (Y = −14.56 + 1.091x; R^2^ = 0.99; *p* = 0.012) and RV (Y = 7.43 + 0.33x; R^2^ = 0.99; *p* = 0.046) sites. The antioxidant activity exerted by the extracts of *A. germinans*, *R. mangle*, and *L. racemosa* varied in a concentration-dependent manner (Table 2, Figure 4), and *L. racemosa*-RV extract (EC_50_: 39.74 ± 0.91 μg/mL) presented the highest antioxidant potency.

**Figure 5:**
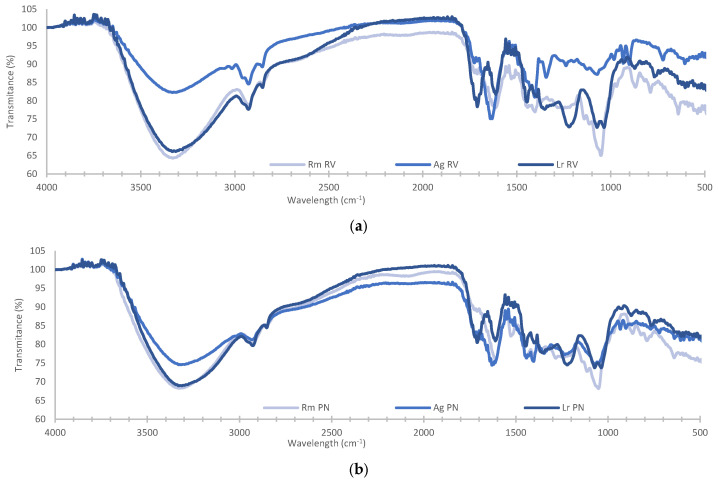
FTIR spectra of the methanolic extracts of the leaves of *R. mangle*, *A. germinans*, and *L. racemosa* collected in Río Verde (**a**) and Petén Neyac (**b**) in the RBLP.

FTIR analysis of methanolic extracts of *R. mangle*, *A. germinans*, and *L. racemosa* was used to record variations in the banding pattern in the mid-infrared region (Figure 5). The bands at 3328.78 ± 2.49 cm^−1^ were related to R-OH narrowing in water, alcohols, phenols, flavonoids, and amines were corroborated with the bands at 1370 and 1221 cm^−1^ assigned to phenolic hydroxyls [36,37]. Bands at 2929.38 ± 2.71 and 2851.52 ± 1.32 cm^−1^ corresponded to narrowing and deforming vibrations of methyl and methylene groups [38,39], the band at 1471 cm^−1^ to the bending of methylene [40], and the band at 1402.5 ± 1.36 cm^−1^ to the symmetrical scissor and rocking bending of methyl groups [41]; these bands suggest the presence of lipids in the extracts. Bands at ±1700, 1617, and 1230 cm^−1^ indicated the presence of carbonyl carbons, aliphatic amines (asymmetric stretching of N-H), and esters, respectively [42]. Bands at 1035.8, 1052.2, and 1071.8 cm^−1^ correspond to vibrations of C-O-C bonds, which are related to the presence of heterosides. The band at 982.6 was related to -C = C-H narrowing vibrations [43].

The presence of chlorophylls was confirmed with bands at 1657, 1640, and 1549 cm^−1,^ which are related to carbonyl carbons (C13) of the E ring, to the aldehyde of the B ring and the C = C bonds of the macrocycle [44]. Carotenoids were identified with the bands at 2924, 2854, 1643, and 1510 cm^−1^ [45]. Quercetin was identified with bands at 3248, 1670, 1500, 1000, and 650 cm^−1^ [46], Gallic acid with bands at 1654, 1100, 1025, 763, and 669 cm^−1^ [47], and caffeic acid with bands at 1645, 1625, 1530, 1450, 1280, 1217, 1120, 815, and 648 cm^−1^ [48].

**Figure 6:**
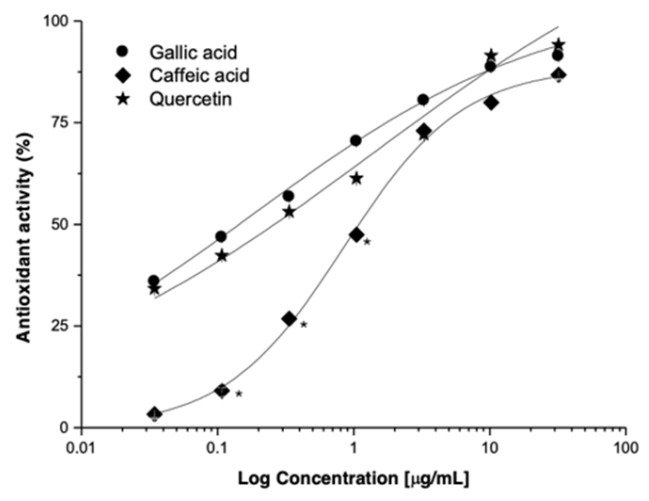
Antioxidant activity induced by quercetin (Q), gallic acid (GA), and caffeic acid (CA); results expressed as the average ± SD of three experiments with three replicates each. (* *p* < 0.05 vs. Q).

The participation of caffeic acid (CA), gallic acid (GA), and quercetin (Q) in the antioxidant activity was evaluated in the DPPH model. CA, GA, and Q exert an antioxidant activity in a concentration-dependent manner (Figure 6, Table 3). The potency of Q was >300 times higher than *L. racemosa*-RV extract, and the efficacy increased by ±25%. It is important to highlight the antioxidant activity exerted by GA and CA (*p* < 0.05).

## 4. Discussion

Previous studies indicate that methanolic extracts from different mangrove species (*R. mangle*, *A. germinans*, and *L. racemosa*) exert a concentration-dependent antioxidant effect [49]. However, the mangrove is a community susceptible to changes in precipitation, temperature, and salinity, among other factors, and these are reflected in photosynthetic responses [50], in the production of leaf litter, hypocotyls [51], propagules, leaves, and flowers [2], among other phenotypic manifestations. Factors such as phenological development, seasonality, time, temperature, polarity of solvents, and extraction methods, among other aspects, influence the raw materials obtained from wild species.

In Río Verde (RV), mesohaline and hypoxic–oxic conditions are observed in the forest along the coastline. Further inland, the forest exhibits euhaline and hypoxic–anoxic conditions. In Petén Neyac (PN), mesohaline and oxic environments are recorded in the coastal forest, while further inland, euhaline and hypoxic conditions are observed. These conditions regulate the physiological processes that allow adaptation to salinity and capture atmospheric oxygen, that are reflected in changes to the forest’s structure and functionality [52]. Hence, it is important to understand the plants’ metabolism, before and after their harvest.

The gravimetric analysis of the extracts derived from samples collected in RV and PN allows the measurement of changes in the amount of extractable material from the leaves of *A. germinans*, *R. mangle*, and *L. racemosa*. Qualitative phytochemical analysis is used to characterize phytochemicals [53] to identify various families of secondary metabolites. However, chemical reactions do not achieve chemical differentiation.

UV-Vis spectroscopy showed absorption changes in the regions of λ: 400–450 and λ: 640–690 nm, which are related to carotenoids and chlorophylls [54]. The peak between 410–430 nm is related to chlorophyll (Abs: 410, 430, and 662 nm) and to degradation products such as chlorophyllide (Abs: 412, 431, and 662 nm; loss of phytol) and pheophytin (Abs: 410 nm; Mg^2+^ loss) [55]. The absence of the shoulder at λ: 430 nm in the *L. racemosa* spectrum supports differences in the chlorophyll degradation process that can be extrapolated to the metabolic content. In this context, analysis is required under other experimental conditions that avoid degradation and guarantee the presence of pigments [56], among other chemical entities.

The chromatographic profile confirmed the complexity of the extracts. *L. racemosa* presented the greatest metabolic diversity, compared to *A. germinans* and *R. mangle.* Gallic acid, caffeic acid, and quercetin were detected at 272 nm. Other phenolic acids (methyl gallate, syringic acid, *p*-coumaric acid, synaptic acid, and ferulic acid) and flavonoids (catechin, rutin, myricetin, apigenin, and kaempferol) have also been detected at that wavelength and showed different retention times [30]. In this context, salinity had a favorable impact on the biosynthesis of gallic acid, among other metabolites of a phenolic nature (*L. racemosa*-RV vs. *L. racemosa*-PN; Figure 3). Future analytical experiments (e.g., HPLC-MS) are required to confirm the presence and abundance of these metabolites. The analysis of the functional group and fingerprint region of the FTIR spectrum allows us to confirm the variation of the chemical content of the samples under study.

The variations in extraction yield and the estimation of chlorophyll a/b are related to the stomatal system and photorespiration, due to the differences in the photosynthetic adaptations of *R. mangle*, *A. germinans*, and *L. racemosa*. The leaves of *L. racemosa* are amphistomatic, with high stomatal density (86.1 and 55.3/mm^2^ upper and lower) and structural and organizational differences in the guard cells [57,58], in hypostomatic leaves of *R. mangle* (74.2 stomata/mm^2^) and *A. germinans* (86.1 stomata/mm^2^), suggesting better management of salinity through control of stomatal opening, carbon gain and instantaneous water use efficiency (WUE) [59]. Situations that modify the flow of O_2_, CO_2_, water, the Calvin cycle, and production of 3-phosphoglycerate to be used in central metabolic pathways [60].

Salinity is an abiotic stressor and activator of the shikimate pathway, example of a metabolic defense pathway in the presence of reactive oxygen species [61]. Aromatic amino acids, cinnamic acids, and their derivatives result from the shikimate pathway, metabolites recognized for their antioxidant properties, among other health benefits [62]. An increase in the content of phenols and flavonoids has been reported in plants (e.g., *Lepidium sativum*, *Crocus sativus*, and *Schizonepeta tenuifolia*) subjected to salt stress conditions [63,64,65]. In the context of the Campeche mangrove, it is important to establish the experimental basis regarding the impact of salinity on the metabolic profile, to understand the physiological, ecological, and phytochemical mechanisms that are applicable to the search for new drugs. Pharmacological effects will depend on chemical content, the abundance of metabolites, dosage, and frequency of use.

Many effects induced by natural products (NP) are consistent with Concentration-Response Curves (CRC). This model allows the determination of the pharmacological parameters of potency (EC_50_) and efficacy (E_max_) induced by the extracts, metabolites, and positive controls. Extracts and NP derived from mangrove species induce concentration-dependent antioxidant effects, and the antioxidant effect of *L. racemosa*-RV is three times more potent than that of *L. racemosa*-PN (Table 2). The antioxidant effect of NP is proportional to NP-DPPH complex concentration. The maximum effect is reached when an NP occupies DPPH molecules. The shape and shift of CRCs are related to the disparity in the affinities of the NP to donate electrons to neutralize the DPPH radical. CA and Q can be used as biomarkers of *L. racemosa*-RV standardized extracts and will contribute to the safe and effective use of the region’s plants.

Changes in antioxidant activity are related to biological variability, the collection site, post-harvest processes (e.g., drying), handling (light, temperature, extraction, and storage), and other oxidative conditions [66]. Antioxidant activity depends on hydroxylation patterns in aromatic systems, carbonyl carbons (aromatic acids, esters, or lactones), and methyl esters of phenolic acids’ presence [67]. Also, the Folin–Ciocalteu reaction (FCR) is not specific to phenolic compounds, as the FCR reduces and reacts with various non-phenolic compounds [68]. Gallic acid was found to be one of the most abundant and important metabolites in the induction of the antioxidant effect by methanolic extract of *L. racemosa*-RV. However, other metabolites with antioxidant activity have also been observed. This opens the opportunity for the identification of other active metabolites from *L. racemosa*-RV, as well as evaluation of the synergy in the induction of antioxidant activity. Therefore, more information is required regarding the analytical phytochemical profile of mangrove species for later use in the physiological, ecological, and pharmacological contexts, among others.

## 5. Conclusions

Through an experimental matrix (two sites, three species) that generates chemical, chromatographic, and spectroscopic information, the present work documents for the first time the influence of salinity on *R. mangle*, *A. germinans*, and *L. racemosa* grown in Río Verde and Petén Neyac, RBLP, Campeche, México. In this context, MeOH extracts of *L. racemosa* (RV) leaves are well-suited for a bioguided phytochemical study to provide valuable insights into their medicinal properties, promote ecological conservation, and stimulate innovation across various industries. GA is the main metabolite affected by salinity and participates in antioxidant activity, which could explain the physiological adaptations of *L. racemosa* and its traditional uses. However, other metabolites could act synergistically in situations of physiological stress. Further analytical studies are needed to confirm the impact of salinity on the biosynthesis of secondary metabolites, as well as phytochemical and pharmacological studies that allow the development of standardized extracts from *L. racemosa*-RV, among other value products.

## Figures and Tables

**Table 1:** Extraction yield and pigment content in the methanolic extracts of leaves of *R. mangle* (*Rm*), *L. racemose* (*Lr*), and *A. germinans* (*Ag*).

Species	Site	Salinity (PSU)	Yield (%)	Chlorophyll				Carotenoids (mg/g)
				A (mg/g)	B (mg/g)	Ratio a/b	Total (mg/g)	
*Ag*	RV	74.87 ± 5	21.98 ± 0.94	0.16 ± 0.06	0.07 ± 0.03	2.3	0.23 ± 0.05	0.01 ± 0.0030
	PN	58.93 ± 3	18.04 ± 2.07	0.12 ± 0.03	0.05 ± 0.01	2.4	0.17 ± 0.04	0.008 ± 0.0010
*Rm*	RV	42.12 ± 4	17.46 ± 0.46	0.26 ± 0.02	0.10 ± 0.01	2.6	0.35 ± 0.02	0.018 ± 0.0004
	PN	42.25 ± 2	21.52 ± 2.22	0.17 ± 0.02	0.06 ± 0.01	2.8	0.23 ± 0.03	0.013 ± 0.0010
*Lr*	RV	43.25 ± 4	35.21 ± 0.76	0.09 ± 0.03	0.01 ± 0.01	9	0.10 ± 0.04	0.005 ± 0.0040
	PN	40.59 ± 2	35.38 ± 0.14	0.11 ± 0.04	0.02 ± 0.01	5.5	0.13 ± 0.05	0.009 ± 0.0020

**Table 2:** Phenols, flavonoids, and antioxidant activity (DPPH) induced by extracts from *A. germinans* (Ag), *R. mangle* (Rm), *L. racemosa* (Lr). RV: Río Verde; PN: Petén Neyac.

Species	Site	Phenols (mg/g)	Flavonoids (mg/g)	Potency EC_50_ (μg/mL)	Efficacy E_max_ (%)
*Ag*	RV	0.42 ± 0.2	0.26 ± 0.08	157.83 ± 5.44	61.44 ± 3.45
	PN	0.63 ± 0.3	0.20 ± 0.08	194.76 ± 16.98	68.75 ± 12.5
*Rm*	RV	0.64 ± 0.2	0.14 ± 0.04	59.81 ± 5.35	74.42 ± 8.95
	PN	0.86 ± 0.3	0.23 ± 0.07	294.47 ± 14.19	53.69 ± 9.17
*Lr*	RV	0.54 ± 0.3	0.15 ± 0.02	39.74 ± 0.91	67.82 ± 1.00
	PN	0.75 ± 0.2	0.16 ± 0.13	123.47 ± 4.87	73.27 ± 4.56
*C. sinensis*	-	-	-	8.69 ± 0.34 *	92.36 ± 0.87 *

* *p* < 0.05 vs. *C. sinensis*.

**Table 3:** Antioxidant activity induced by quercetin, caffeic acid, and gallic acid in the DPPH model.

Activity	Caffeic Acid	Gallic Acid	Quercetin	*L. racemosa*-RV
EC_50_ (μg/mL)	1.09 ± 0.06	0.22 ± 0.01	0.12 ± 0.01 *	39.74 ± 0.91
E_max_ (%)	86.75 ± 0.20	91.35 ± 0.14	94.15 ± 0.11 *	67.82 ± 1.00
Relative Potency	−9.08	−1.83	1	−331.2
Relative Efficacy	91.1	97.0	100	72.0

* *p* < 0.05 vs. *L. racemosa* (RV).

## Data Availability

All data generated and analyzed in this work are included in this manuscript.

## Supplementary Materials

The following supporting information can be downloaded at: https://www.mdpi.com/article/10.3390/plants14050800/s1, Figure S1 Chromatogram profile obtained by HPLC of gallic acid (GA; t = 5.48 min) and quercetin (Q; t = 24.6 min) at λ = 272 nm. Mobile phase: Sol A (H_2_O-acetic acid 1%), Sol B (ACN 100%); 10–40% (0–28 min): 40–60% (28–39 min); 60–90% (39–50 min). Flux: 0.5 mL/min.
