# Supplementary material for: Salinity as an Inducer of Antioxidant Activity Exerted by Mangrove Species from Campeche, Mexico
Source: Plants (Basel). 2025 Mar 4;14(5):800. doi: 10.3390/plants14050800 (PMC11902564; doi:10.3390/plants14050800)
Supplement: Supplementary file 1 [file plants-14-00800-s001.zip › plants-3307874-supplementary.pdf]

plants

MDPI

SUPPLEMENTARY MATERIAL

Salinity as an inducer of antioxidant activity exerted by mangrove species from Campeche, Mexico

Carlos A. Chan-Keb [1], José L. Aragón-Gastélum [1], Claudia M. Agraz-Hernández [2], Roman A. Pérez-Balan [1], Eduardo J. Gutiérrez Alcántara [1], Marco A. Popoca-Cuaya [1], Mónica A. Guillen-Poot [3], Emanuel Hernández-Núñez [4] and Francisco J. Aguirre-Crespo[1,*]

1. Facultad de Ciencias Químico-Biológicas, Universidad Autónoma de Campeche, San Francisco de Campeche, Campeche, México. Av. Agustín Melgar S/N entre Calle 20 y Juan de la Barrera. Col. Buenavista. CP 24039. fjaguirr@uacam.mx, jlaragon@uacam.mx, mapopoca@uacam.mx, roaperez@uacam.mx, carachan@uacam.mx
2. Instituto EPOMEX, Universidad Autónoma de Campeche, San Francisco de Campeche, Campeche, México. Av. Agustín Melgar S/N entre Calle 20 y Juan de la Barrera. Col. Buenavista. CP 24039. clmagraz@uacam.mx
3. Independent Researcher moniguillen7@gmail.com
4. Departamento de Estudios de Posgrado e Investigación del Instituto Tecnológico Superior del Calkiní en el Estado de Campeche (ITESCAM), Av. AH Canun S/N San Felipe, Calkiní, Campeche, México. CP 24900, ehernandez@itescam.edu.mx

* Correspondence: fjaguirr@uacam.mx

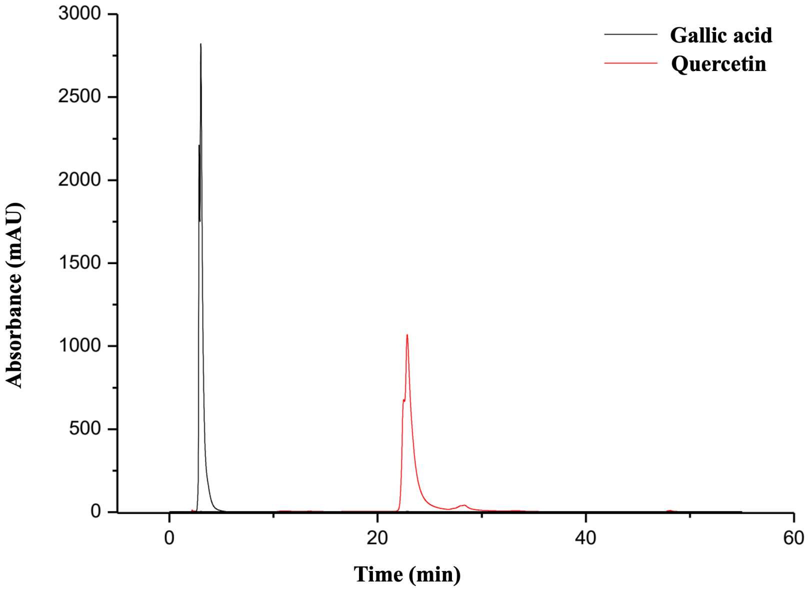

Figure S1. Chromatogram profile obtained by HPLC of gallic acid (GA) and quercetin (Q) a 272 nm. Mobile phase: Sol A (H_2O-acetic acid 1%), Sol B (ACN 100%); 10-40% (0-28 min): 40-60% (28-39 min); 60–90% (39-50 min). Flux: 0.5mL/min.

References

1. Seal, T. Quantitative HPLC analysis of phenolic acids, flavonoids, and ascorbic acid in four different solvent extracts of two wild edible leaves, *Sonchus arvensis* and *Oenanthe linearis* of North-Eastern region in India. *J. Appl. Pharm. Sci.* **2016**, *6*(2), 157-166. http://dx.doi.org10.7324/JAPS.2016.60225
2. Cu-Quiñones, L.D. Desarrollo de un prototipo de forma farmacéutica a partir de las hojas de *Jatropha gaumeri* Greenm (pomol che◎). Master's Thesis. 2021, Universidad Autónoma de Campeche.
